# Control of the Induced Handedness of Helical Nanofilaments Employing Cholesteric Liquid Crystal Fields

**DOI:** 10.3390/molecules26196055

**Published:** 2021-10-06

**Authors:** Ju-Yong Kim, Jae-Jin Lee, Jun-Sung Park, Yong-Jun Choi, Suk-Won Choi

**Affiliations:** Department of Advanced Materials Engineering for Information & Electronics, and Integrated Education Institute for Frontier Science & Technology (BK21 Four), Kyung Hee University, Yongin 17104, Gyeonggi-do, Korea; kjuyong0818@naver.com (J.-Y.K.); jjking1443@naver.com (J.-J.L.); pjs426@khu.ac.kr (J.-S.P.); cyj001315@khu.ac.kr (Y.-J.C.)

**Keywords:** chirality, bent-core molecules, helical nanofilaments, cholesteric liquid crystals, chiral dopants, circular dichroism

## Abstract

In this paper, a simple and powerful method to control the induced handedness of helical nanofilaments (HNFs) is presented. The nanofilaments are formed by achiral bent-core liquid crystal molecules employing a cholesteric liquid crystal field obtained by doping a rod-like nematogen with a chiral dopant. Homochiral helical nanofilaments are formed in the nanophase-separated helical nanofilament/cholesteric phase from a mixture with a cholesteric phase. This cholesteric phase forms at a temperature higher than the temperature at which the helical nanofilament in a bent-core molecule appears. Under such conditions, the cholesteric liquid crystal field acts as a driving force in the nucleation of HNFs, realizing a perfectly homochiral domain consisting of identical helical nanofilament handedness.

## 1. Introduction

If a molecule (or superstructure) cannot be superimposed onto its mirror image, it is referred to as chiral. Ever since the enantiomers of tartaric acid were resolved by Pasteur [1], chirality has been a remarkable subject in the fields of chemistry, physics, biology, pharmacy, and material science. In the field of liquid crystal (LC) science, chirality is also a fascinating research topic [2,3,4,5,6,7,8,9] because unique LC phases (such as cholesteric and blue phases) appear by introducing chirality.

**Figure 1:**
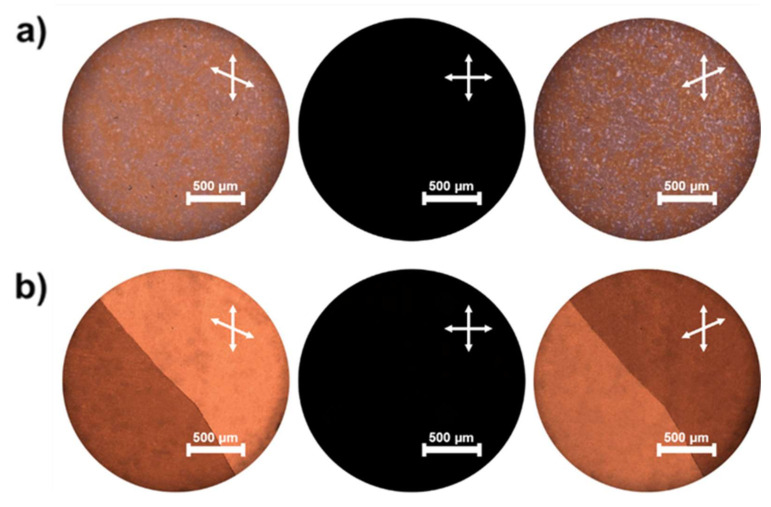
Typical polarized optical microscopy (POM) image in: (**a**) B4 phase of bent-core (BC) molecule (P-9) and in (**b**) B4 phase blended with a rod-like liquid crystal (LC) molecule (5PCB). (Mixing ratio: 55 wt% P-9 and 45 wt% 5PCB).

Since the discovery of chemically achiral bent-core (BC) molecules [10,11] that possess contradictory characteristics such as polarity and chirality, there have been many studies worldwide on unique LC phases originating from BC molecules. The unique LC phases related to BC molecules are different from conventional LC phases observed in rod-like molecules [10]. Among the unique LC phases, the B4 phase is a semi-crystalline phase with hexatic LC ordering [12], in which achiral BC molecules spontaneously form chiral superstructures, i.e., helical nanofilaments (HNFs) [13,14,15,16]. Therefore, the B4 phase is also called the HNF phase. Two spontaneously segregated chiral domains consisting of opposite-handed HNFs are formed in the B4 phase because the BC molecule is achiral. Two chiral domains originating from the two opposite handedness of the helix in the B4 phase are observed by conventional polarized optical microscopy (POM) [13]. The two domains become apparent as bright and dark regions when one of the polarizers is rotated clockwise by a small angle with respect to the crossed position, and the brightness of the two areas is interchanged when the polarizer is rotated counterclockwise. The observed texture exhibits grainy domains segregated into two chiral domains of equal probability, as shown in Figure 1a. Although the grainy domains with a certain chirality can be grown to a certain extent by mixing rod-like LC molecules, as shown in Figure 1b, two different domains with different chiralities appear because the BC molecules are chemically achiral.

Several methods have been reported to overcome this obstacle by controlling the chiral domain consisting of a particular handedness of the helix in the B4 phase. These include doping chiral BC LC analogues [17], the use of a chiral surface [18], irradiation with circularly polarized light using photoisomerizable BC molecules [19], employing a twisted nematic director orientation [20,21], and growth from phospholipid chiral layers [22]. Beyond our expectations, it has been reported that the addition of chiral dopants to BC molecules has no effects on the induced handedness of HNFs in most cases [12,17,23]. This is due to the low solubility of the chiral dopants in the HNFs and the high temperature at which the HNFs appear [12,23].

Herein, we propose a simple and powerful strategy to control the induced handedness of HNFs by employing a cholesteric LC field obtained by a rod-like nematogen doped with a chiral dopant. By blending a BC molecule with a mixture exhibiting the cholesteric phase, which appears at a temperature higher than the temperature at which the HNF in a BC molecule appears, a nanophase-separated HNF/cholesteric phase is formed. Because HNFs of BC molecules are generated under the condition of a pre-formed cholesteric LC field upon cooling, the handedness of HNFs can be perfectly controlled by the twist sense of the chiral dopant used.

## 2. Results and Discussion

**Figure 2:**
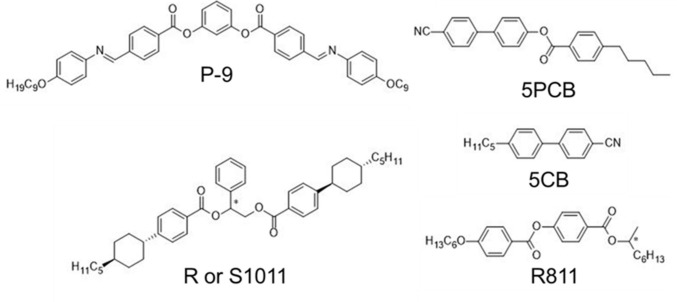
Chemical structures of materials used in this study (bent-core (BC) molecule: P-9; rod-like molecules: 5PCB and 5CB; chiral dopants: R or S1011, R811).

First, we prepared three cholesteric LCs (CLC-1R and two CLC-2 (CLC-2R and CLC-2S)), consisting of rod-like nematogen and chiral dopant (CLC-1R: nematogen (5CB, 97 wt%) and chiral dopant (R1011, 3 wt%); CLC-2R: nematogen (5PCB, 97 wt%) and chiral dopant (R1011, 3 wt%); CLC-2S: nematogen (5PCB, 97 wt%) and chiral dopant (S1011, 3 wt%)). Furthermore, 5CB, R, and S-1011 were purchased from Sigma-Aldrich, and 5PCB was synthesized by our group. CLC-1R and CLC-2R have the same chiral dopant but different host nematogens. CLC-2R and CLC-2S show the same helical pitch but an opposite helical twisting direction because blended chiral dopants (R1011 and S1011) show opposite chiral senses. The isotropic-to-cholesteric transition temperatures (T_ch_) of the CLC-1R and CLC-2 series were 30 °C and 190 °C, respectively. Next, the CLC-1R and CLC-2 series were blended with the BC molecule (P-9, synthesized by our group). The transition temperature (T_B4_) at which the HNF appeared in P-9 was 140 °C. The mixing ratios of cholesteric LC and BC molecules were 45 and 55 wt%, respectively. The T_ch_ of CLC-1R, at which the cholesteric phase appeared, was lower than T_B4_ at which the HNF appeared. In contrast, T_ch_ of the CLC-2 series was higher than that of T_B4_ of P-9. The chemical structures used in this study are shown in Figure 2.

**Figure 3:**
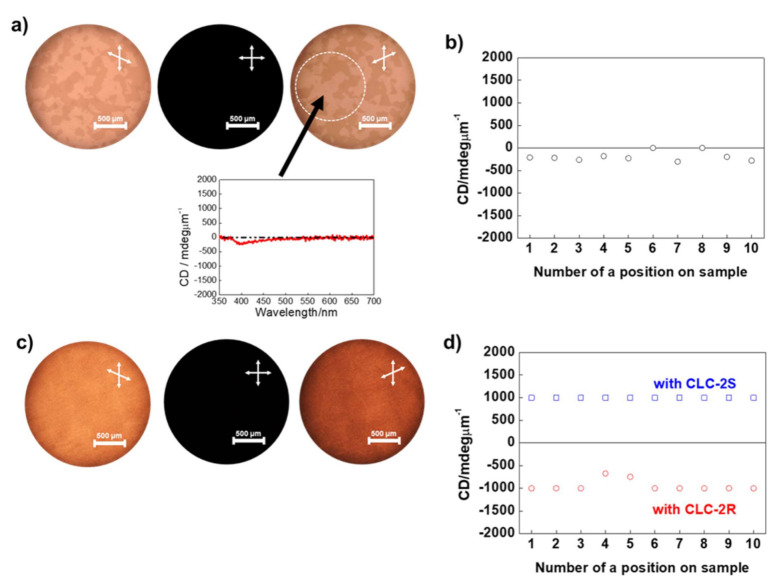
(**a**) Typical polarized optical microscopy (POM) images and typical circular dichroism (CD) spectrum observed on a sample position. CD signals are detected from a spot of 1.2 mm diameters. (**b**) CD intensities (at ~380 nm) at 10 different positions on sample of <helical nanofilament (HNF)/cholesteric> state in P-9 doped with CLC-1R. (**c**) Typical POM images, and (**d**) CD intensities (at ~380 nm) at 10 different positions on sample of <HNF/cholesteric> state in P-9 doped with CLC-2R and CLC-2S.

In mixtures where BC molecules serve as the host, it is well known that blending of mesogenic guests leads to phase separation, in which the guest is expelled into the empty spaces between the HNFs in the B4 phase [3,4,5,12,23,24,25]. In our case, the cholesteric phase is expelled into the empty spaces between the HNFs; thus, hereafter, we refer to this phase-separated phase as the <HNF/cholesteric> state. Figure 3a shows the typical POM images of the <HNF/cholesteric> state in P-9 doped with CLC-1R and the typical circular dichroism (CD) spectrum observed on a sample position in which the sign of the CD intensity at ~400 nm is negative. By rotating the polarizer or analyzer from the cross-polarization position, we can distinguish two clearly different domains with opposite optical rotations in the <HNF/cholesteric> state of P-9 doped with CLC-1R. Figure 3b shows the CD intensity at ~400 nm, depending on the sample position, for 10 observations. The evaluated CD intensity was small because two chiral domains, with different handedness of the HNF, exist in a detected spot of 1.2 mm diameters. CD analysis was performed using JASCO J-815. POM and CD results indicated that there were no effects on induced handedness of HNFs in the case of blending CLC-1R into P-9.

In contrast, as shown in Figure 3c, in the <HNF/cholesteric> state of P-9 doped with CLC-2R, two clearly different domains with opposite optical rotations could not be distinguished, indicating that one chiral domain possessing a uniform handedness of HNF could be formed across the sample. This was also supported by CD analysis. Large CD intensity with negative sign at ~400 nm was evaluated irrespective of the sample position during 10 observations, as shown in Figure 3d. We also performed CD analysis in the <HNF/cholesteric> state of P-9 doped with CLC-2S. Interestingly, large CD intensity with positive signs was always observed irrespective of the sample position during 10 observations. At 350–450 nm in the <HNF/cholesteric> state, *R*-chiral dopant systems (*R*1011) induced large CD signals with negative signs, whereas *S*-chiral dopant systems (*S*1011) induced large CD signals with positive signs without exception. These results strongly indicate that a mono domain with uniform handedness of HNF can be realized by blending the CLC-2 series, and the chiral sense of the HNF can also be controlled by the sense of the chiral dopant.

The CLC-2 series influenced the induced handedness of HNFs, but CLC-1R did not because the T_ch_ of the CLC-2 series was higher than the T_B4_ of the host BC molecule. Under the condition of a pre-formed cholesteric LC field on cooling, HNFs of BC molecules are nucleated. Thus, the handedness of HNFs can be effectively influenced by that of the cholesteric LC field. On the other hand, if T_ch_ is lower than T_B4_, the cholesteric LC field does not influence HNF nucleation because HNF is generated in advance before the cholesteric LC field is generated during cooling. The chiral strength of the isotropic liquid doped with chiral dopants was much smaller than that of the condensed liquid crystalline phase doped with chiral dopants. The chiral field exerted by the chiral fluid was not enough to determine the handedness of HNFs; thus, CLC-1R did not influence the handedness of the HNFs.

**Figure 4:**
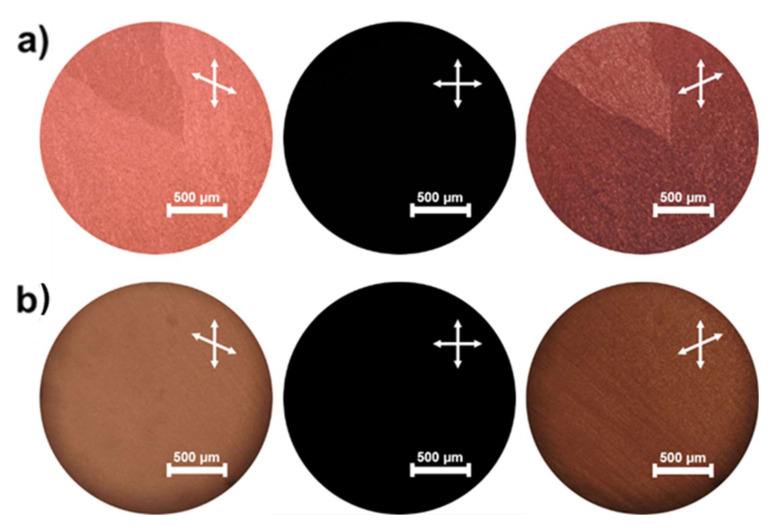
Typical polarized optical microscopy (POM) images of <helical nanofilament (HNF)/cholesteric> state in P-9 doped with (**a**) CLC-3 and (**b**) CLC-4.

Finally, we prepared two other cholesteric LCs (CLC-3 and CLC-4) consisting of 5PCB and chiral dopant R811 (Sigma-Aldrich) (CLC-3: nematogen (5PCB, 97 wt%) and chiral dopant (R811, 3 wt%), CLC-4: nematogen (5PCB, 85 wt%) and chiral dopant (R811, 15 wt%)). The T_ch_ values of CLC-3 and CLC-4 were 190 °C and 180 °C, respectively, upon cooling. The T_ch_ of both cholesteric LCs is higher than T_B4_ of BC molecules (P-9). CLC-3 and CLC-4 were blended with 60 wt% BC. Figure 4a,b show POM images of the <HNF/cholesteric> state in P-9 doped with CLC-3 and CLC-4, respectively. Although two different chiral domains were still observed in the <HNF/cholesteric> state of P-9 doped with CLC-3, perfectly uniform domains with homochirality could be recognized in the <HNF/cholesteric> state of P-9 doped with CLC-4. In this case, by increasing the amount of chiral dopant R811, a uniform domain could be realized. It is notable that the helical twisting power (HTP) of R811 was approximately one-third of that of R1011 previously used [26]. Thus, to obtain a perfectly uniform chiral domain, the doping amount of R811 requires more than the doping amount of R1011. This result supports that the HTP of the chiral dopant used is also an important factor in controlling the handedness of HNF in BC molecules.

## 3. Conclusions

We succeeded in controlling the handedness of HNF using a cholesteric LC field obtained by a rod-like nematogen doped with a chiral dopant. We obtained homochiral HNF domains in the nanophase-separated HNF/cholesteric phase from a mixture with a cholesteric phase, which appeared at a higher temperature than the temperature at which the HNF in a BC molecule appeared. Under the circumstance of a pre-formed cholesteric LC field on cooling, HNFs of BC molecules are nucleated; thus, the cholesteric LC field effectively influences the nucleation of HNFs. The chiral sense of the nucleated HNF correlates with the chiral sense of the chiral dopant used, which was confirmed by CD observation. The doping amount of the chiral dopant should be optimized according to the HTP of the chiral dopant used. Our proposed method is better than other previously suggested methods because it offers the possibility of easy and universal control of the handedness of HNFs in BC molecular systems.

## Data Availability

The data presented in this study are available on request from the corresponding author.

## Sample Availability

Samples of the compounds are available from the authors.
